# Potential Role of Phytochemicals Against Matrix Metalloproteinase Induced Breast Cancer; An Explanatory Review

**DOI:** 10.3389/fchem.2020.592152

**Published:** 2021-01-15

**Authors:** Yusra Habib Khan, Ambreen Malik Uttra, Sumera Qasim, Tauqeer Hussain Mallhi, Nasser Hadal Alotaibi, Maria Rasheed, Abdulaziz Ibrahim Alzarea, Muhammad Shahid Iqbal, Nabil Khulaif Alruwaili, Salah-Ud-Din Khan, Abdullah Salah Alanazi

**Affiliations:** ^1^Department of Clinical Pharmacy, College of Pharmacy, Jouf University, Sakakah, Saudi Arabia; ^2^College of Pharmacy, University of Sargodha, Sargodha, Pakistan; ^3^Department of Pharmacology, College of Pharmacy, Jouf University, Sakakah, Saudi Arabia; ^4^Institute of Pharmacy, Lahore College for Women University, Lahore, Pakistan; ^5^Department of Clinical Pharmacy, College of Pharmacy, Prince Sattam bin Abdulaziz University, Al-Kharj, Saudi Arabia; ^6^Department of Pharmaceutics, College of Pharmacy, Jouf University, Sakaka, Saudi Arabia; ^7^Department of Biochemistry, College of Medicine, Imam Mohammad Ibn Saud Islamic University, Riyadh, Saudi Arabia

**Keywords:** cancer, breast cancer, phytochemical, matrix metalloproteinase, tumor, Metalloproteinase (MMP), Metalloproteinase (aMMP-8)

## Abstract

World Health Organization (WHO) estimated breast cancer as one of the most prevailed malignancy around the globe. Its incident cases are gradually increasing every year, resulting in considerable healthcare burden. The heterogeneity of breast cancer accounts for its differential molecular subtyping, interaction between pathways, DNA damaging, and chronic inflammation. Matrix metalloproteinases (MMPs) are a group of zinc-containing, calcium dependent endopeptidases which play a substantial role in breast carcinogenesis through several mechanisms. These mechanisms include remodeling of extracellular matrix (ECM), cell proliferation, and angiogenesis which promote metastasis and result in tumor progression. In this context, compounds bearing MMP inhibitory potential can serve as potent therapeutic agents in combating MMPs provoked breast cancer. Current systematic review aimed to encompass the details of potent natural lead molecules that can deter MMPs-provoked breast cancer. Following the critical appraisal of literature, a total of *n* = 44 studies that explored inhibitory effect of phytochemicals on MMPs were included in this review. These phytoconstituents include alkaloids (*n* = 11), flavonoids (*n* = 23), terpenoids (*n* = 7), and lignans (*n* = 2). The most common inhibitory methods used to evaluate efficacy of these phytoconstituents included Gelatin Zymography, Western Blotting, and real time polymerase chain reaction (RT-PCR) analysis. Moreover, current limitations, challenges, and future directions of using such compounds have been critically discussed. This review underscores the potential implications of phytochemicals in the management of breast cancer which could lessen the growing encumbrance of disease.

## Introduction

### Breast Cancer

Breast cancer has been characterized as the most prevalent type of cancer among women across the world. Majority of cancer related deaths observed among women of age ranging from 35 to 55 are due to breast cancer. It has been estimated that one among nine women will suffer from this life threatening breast cancer and about 130 thousand women each year die from this cancer. Invasive breast cancers have been categorized into three different categories based on histological features. These include well-differentiated cancer (Grade 1), moderately differentiated (Grade 2) and poorly differentiated cancer (Grade 3) (Abdulrahman and Rahman, 2012). It is pertinent to mention that primary reason of death from any type of cancer has been attributed to the distant metastasis. Extracellular matrix (ECM) degradation has been considered to be an important attributor for distant metastasis permitting cancer cells to enter local tissue, intra- and extravasate blood vessels and form different metastatic growths. ECM degradation occurs principally due to proteinases secreted by the tumor (Köhrmann et al., 2009). Currently four classes of proteinases are well-defined which includes matrix metalloproteinases, cysteine proteinases, serine proteinases, and aspartic proteinases. All these proteinases collectively contribute to the degradation of ECM. During physiological conditions including ovulation, tissue remodeling, angiogenesis, wound healing, a balance is maintained between proteolytic degradation and regulatory inhibition of proteolysis (Page-McCaw et al., 2007). This physiological balance might be disrupted during the tumor. Matrix metalloproteinases (MMPs) up-regulation has been observed in each type of cancer and found to be associated with the poor prognosis among cancerous patients. Previously conducted studies have linked this up-regulation to an advanced stage of breast cancer, enhanced tumor cells invasion and building of metastatic formations (Duffy et al., 2000).

### Matrix Metalloproteinases

Matrix metalloproteinases (MMPs) belong to zinc dependent endopeptidases, responsible for degradation and remodeling of extracellular matrix (ECM) during organogenesis, wound healing, angiogenesis, apoptosis, cell proliferation, and cancer progression. MMPs (except MP-11) are secreted as inactive zymogens and become activated outside the cell by the virtue of other MMPs or serine proteases (Nagase et al., 2006). MMPs induced ECM degradation facilitates the tumor invasion (Köhrmann et al., 2009). In particular, cancer development through MMPs is dependent on several factors including tumor site, tumor stage, substrate profile, and enzyme localization (Decock et al., 2008). Currently, 24 members of MMPs have been identified in vertebrates, including 23 in human beings. Based on substrate specificity, these MMPs are classified into six broad groups including collagenases (MMP-1,−8,−13,−18), gelatinases (MMP-2,−9), stromelysins (MMP-3,−10,−11), matrilysins (MMP-7,−26), membrane-type (MMP-14,−15,−16,−17,−24,−25), and non-classified MMPs (MMP-12,−19,−20,−21,−23,−27,−28). These different types of MMPs are located in cytosol, subcellular organelles, nucleus, and extracellular regions and serve different role at different stages including cell growth, differentiation, survival, and motility. MMPs induced ECM degradation not only supports tumor invasion but also alter behavior of tumor cell, thereby leads to cancer metastasis and ultimately disease progression. Therefore, inhibition of MMPs activity can serve as a useful therapeutic strategy in combating this life threatening cancer (Yu and Stamenkovic, 2000).

### MMPs Activated Tumorigenic Process in Breast Cancer

MMPs along with directly facilitating tumor invasion by degrading basement membrane also facilitate the release of factors promoting tumor growth or inhibiting apoptosis. Dysregulated MMPs activity facilitates cellular processes leading to DNA damage thus stimulate genomic instability. MMPs play critical roles in the tumor microenvironment. They provide nutrients and oxygen to the growing tumor as well as avenues for metastasis through MMP-mediated blood and lymph vessel formation. They generate tissue disruptive fibrotic stroma through MMP-induced activation of stromal fibroblasts. Moreover, action of MMPs on adipocytes stimulate tumor-promoting metabolism. In addition, phenotypic changes associated with the epithelial-mesenchymaltransition (EMT) are also induced by the direct action of MMPs. The EMT is a developmental process which activates during the tumor progression (Radisky and Radisky, 2015).

**Figure 1:**
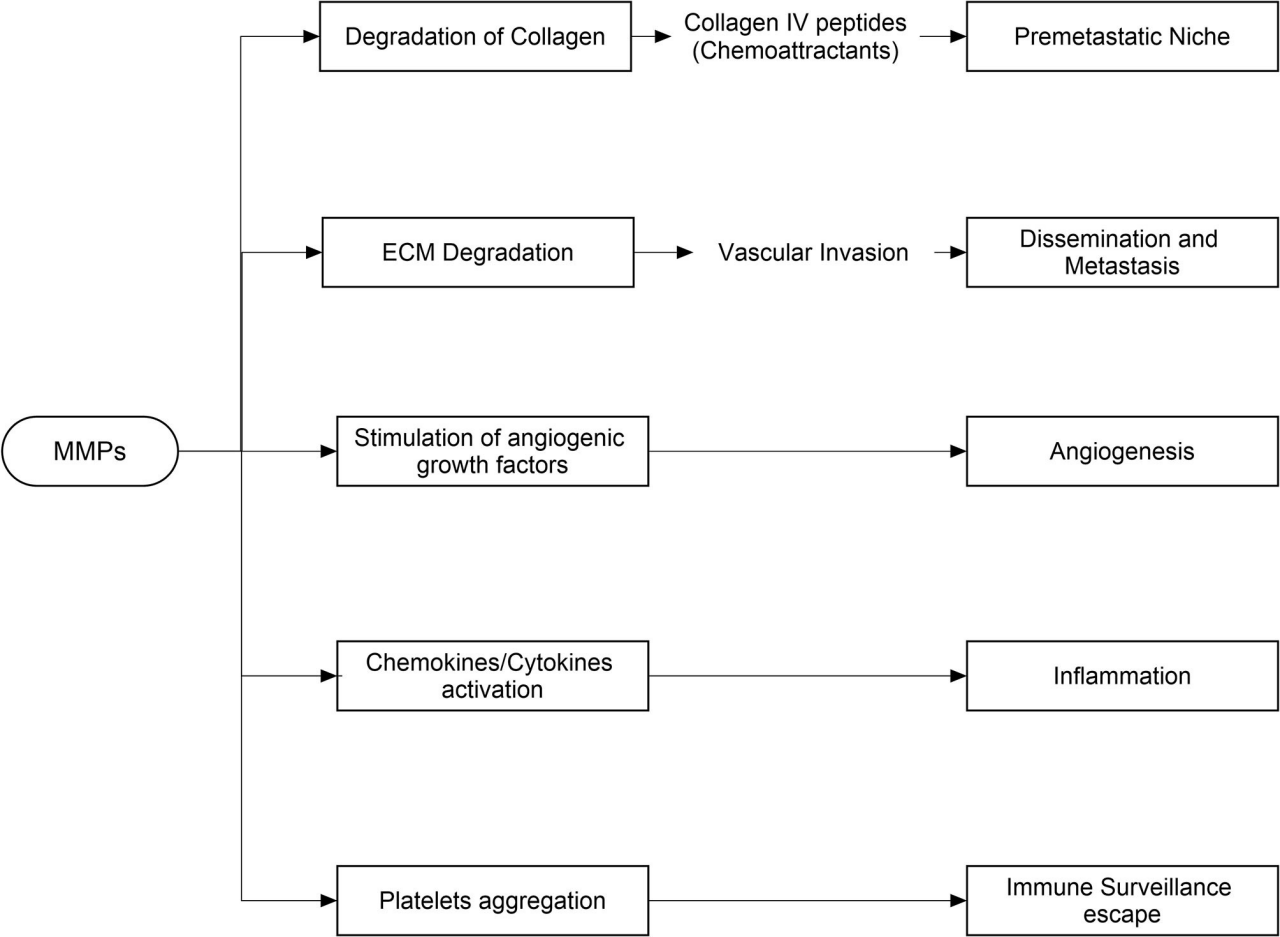
Induction of cancer through matrix metalloproteinase.

The expression of MMPs (MMP-1,−2,−8 to−13,−15,−19,−23,−24,−27, and−28) is comparatively much stronger in cancer tissues than normal tissues of the breast. Degradation of physical barriers during invasion of cancer cell at distant location is regulated by proteolytic activity of MMPs. Invasion is promoted by localization of MMPs to specialized cell surface structure called invadopodia (Weaver, 2006). MMP-1,−2,−3,−9, and−14 are primarily implicated as contributing factors in tumor invasion, metastasis and angiogenesis. Moreover, the progression of breast cancer is attributed to MMP-2,−7,−9,−10,−11,−13,−14, and−15 (Weaver, 2006). MMPs contribute to tumor cell proliferation through release of insulin-like growth factors (IGFs) and epidermal growth factor receptor (EGFR) that promote proliferation. Cancer cell proliferation is linked with the MMP-1,−2,−7, and−9, whereas breast tumor metastasis is linked with the MMP-1,−2,−3,−7, and−9 to−18. Furthermore, owing to its tendency to dissolve in bone matrix, MMP-13 stays in breast bone and contributes breast-bone metastasis. Moreover, the anti-apoptotic effect is regulated by activation of serine/threonine kinase Akt/protein kinase B through signaling cascades of EGFR and IGFR (Gialeli et al., 2009). Furthermore, available evidences support the pivotal role of MMPs−1,−2,−7, and−9 in the tumor angiogenesis (Benson et al., 2013). A brief description of induction of cancer via matrix metalloproteinase has been summarized in Figure 1.

### Inhibitors of Matrix Metalloproteinases

The tissue inhibitors of metalloproteinases (TIMPs) regulate the MMPs activity. Currently, four distinct endogenous specific inhibitors of MMPs have been cloned and sequenced. They include TIMP-1,−2,−3, and−4. TIMPs have high affinity toward active MMPs and inhibit protease activity by forming complex. TIMP-1 has affinity for pro-MMP-9 resulting in complex formation. TIMP-2 possesses affinity for precursor of MMP-2. It is important to note that few TIMPs perform various functions at the same time, thus exhibiting multifunctional properties. In this context, TIMP-1 and TIMP-2 are able to stimulate the cell proliferation at least *in vitro* in addition to the inhibitory action for MMP activities. Moreover, studies have demonstrated the inhibitory effects of TIMP-1 and TIMP-2 against apoptosis (Duffy et al., 2000).

## Methodology

This review is a systematic search of literature with specific inclusion and exclusion criteria. All the published scholarly manuscripts describing the predefined objectives and published in English are included in the current review. However, opinions, perspectives, commentaries, viewpoints, case studies, and manuscript in language other than English are excluded from the review. PubMed, Scopus, Google Scholar, SciFinder, Natural Products Updates (NPU), Scientific Electronic Library Online (SciELO), ScienceDirect, Cochrane Library, and SciVerse were utilized for the literature search. Literature search was subjected without any time limit in order to include all relevant information from the date of inception. The following descriptors were used with various Boolean operators: Flavonoids; Alkaloids; Phytochemicals; Chemical structure; Breast cancer; MMPs; metalloproteinases; Matrix metalloproteinases; Tumor; Metastasis; Tumorigenic process; TIMPs; Lignans; Glycosides; Terpenoids. The process of data screening was initiated in January 2020. All the searched articles were based on the abstract analysis. Full texts of the selected article were retrieved and 44 manuscripts were selected following the critical appraisal. All the relevant data was extracted from the articles and tabulated. The information tabulated includes phytochemicals including alkaloids, flavonoids, lignans, glycosides, and terpenoids. For each phytochemical, name of compound, its source, chemical structure, inhibitory mechanism, assay method, and relevant reference were included in the Table 1.

**Table 1:** Phytochemicals modulating MMP activity by inhibiting its activation and associated signaling pathways.

**Compounds name**	**Source**	**Structure**	**MMP inhibitory mechanism**	**IC50 value**	**Analysis technique employed**	**References**
**Alkaloids**						
Berberine	*Berberis vulgaris*	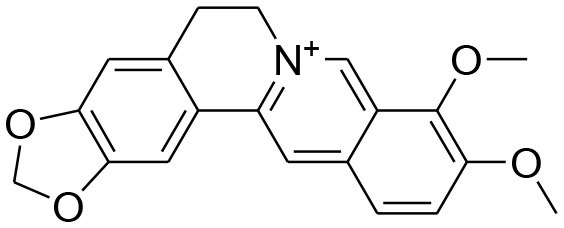	Downregulation of MMP-2/-9 expression	25μM	RT-PCR analysis Gelatin zymography	Kim et al., 2018a,b
			Attenuation of TNF-α induced expression of MMP-9		Gelatin zymography Western blotting	Kim et al., 2008
			Reduced mRNA expression of MMP2/-9 via modulatingAkt signaling pathway		Gelatin zymography RT-PCR analysis Western blotting	Kuo et al., 2012
Evodiamine	*Evodia rutaecarp*	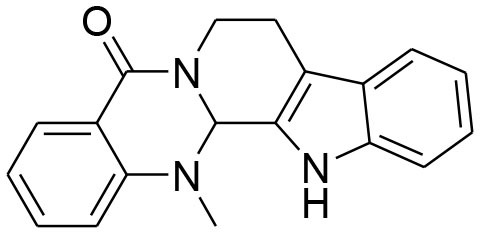	Suppressed expression of MMP-9	6 μM	Western blotting	Du et al., 2013
Matrine	*Sophoraalopecuroides*	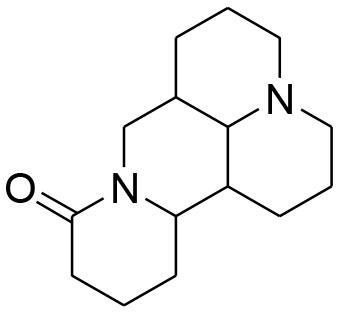	Suppression of invasion and activation of MMP-9/−2 along with attenuated gene expression of MMP-9/−2	0.8 μM	Gelatin zymography RT-PCR analysis	Yu et al., 2009
Piperine	*Piper nigrum*	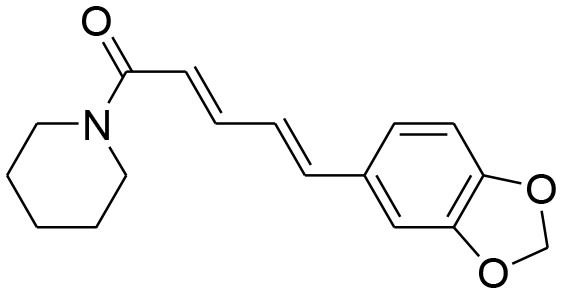	Suppressed MMP-9 and MMP-13 expression	78.5 μM	RT-PCR analysis	Lai et al., 2012
			Suppression of mRNA expression level of MMP-2 /-9		RT-PCR analysis	Do et al., 2013
			Suppression of EGF-induced MMP-9 activation		Transfection and luciferase assay	
Sanguinarine	*Sanguinariacanadensis*	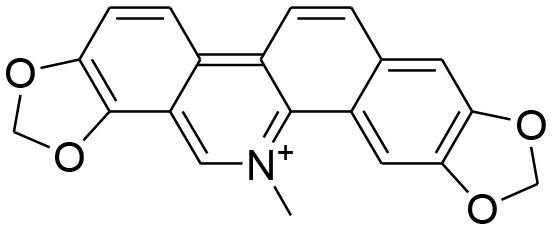	Suppression of MMP-2/-9 expression dose dependently	5.2 μM	Western blot analysis RT-PCR analysis Gelatin zymography	Choi et al., 2009
			Suppression of MMP-9 expression by inhibiting NF-κB and AP-1 signaling pathway		Chromatin immunoprecipitation (ChIP) assay and Transient transfection and dual luciferase assay	Park et al., 2014
α-tomatine	*Lycopersiconpimpinellifolium*	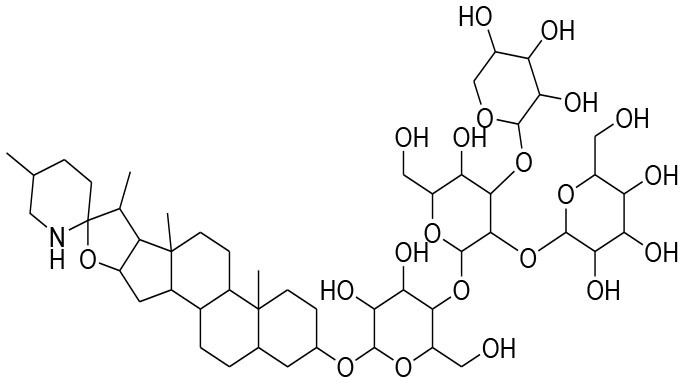	Inhibition of TPA-provokedMMP-2/-9 expression, invasion and migration via inhibition of ERK phosphorylation in MCF-7 Cells	7.07 μM	Gelatin Zymography RT-PCR analysis Western blotting	Shi et al., 2013
Lycorine	*Narcissus tazetta*	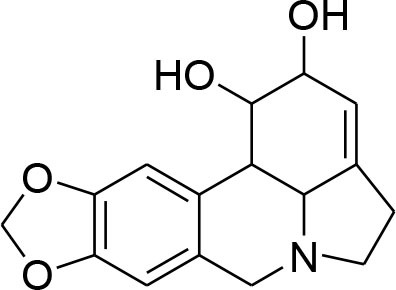	MMP-2/-9 down regulation	7.5 μM	Western blotting	Wang et al., 2017
**Flavonoids**						
Curcumin	*Curcuma longa*	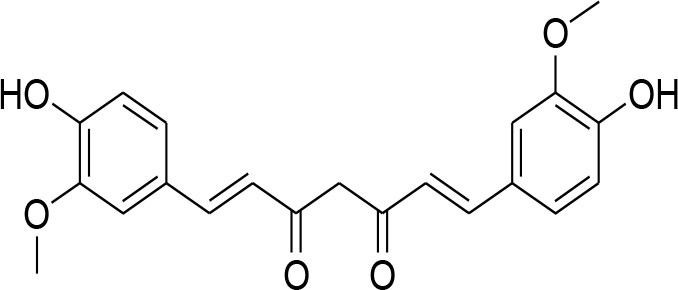	TPA-instigated MMP-9 expression inhibition and transcriptional activation of MMP-9 in MCF-7 cell	6 μM	RT-PCR analysis Western blotting Gelatin zymography Electrophoretic mobility shift assay (EMSA)	Hassan and Daghestani, 2012
			MMP-2/-9 down regulation		RT-PCR	Kim et al., 2012
Epigallocatechin	*Camellia sinensis*	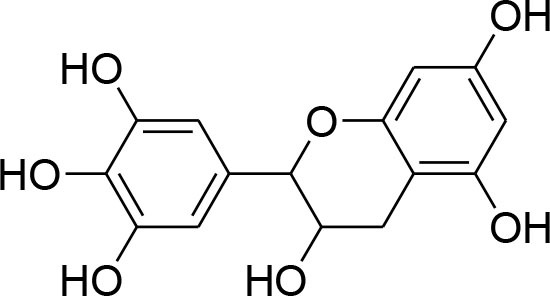	Reduced activity, protein and mRNA MMP-9 Expression via inhibition of NF- κB and AP-1 signaling pathway Overexpression of tissue inhibitor of MMP 1 (TIMP-1)	27.12 μM	Gelatin zymography, western blotting RT-PCR analysisELISA Electrophoretic mobility shift assay (EMSA)	Sen et al., 2010
Genistein	*Glycine max*	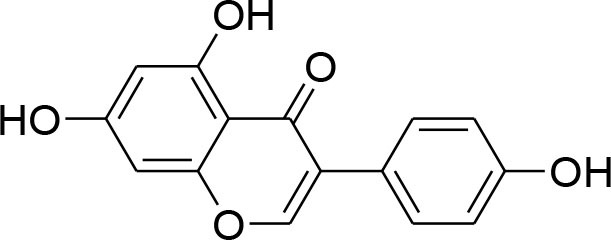	Down-regulation of all MMP genes [(MMP-1,−2,−3,−7,−9,−11,−14,−15,−16)] Up regulation of TIMP-1 level while TIMP-2 level was suppressed in MDA-MB-231 and MCF-7 cell		RT-PCR analysis Gelatin zymography	Kousidou et al., 2005; Lee et al., 2007
Kaempferol	*Moringaoleifera*	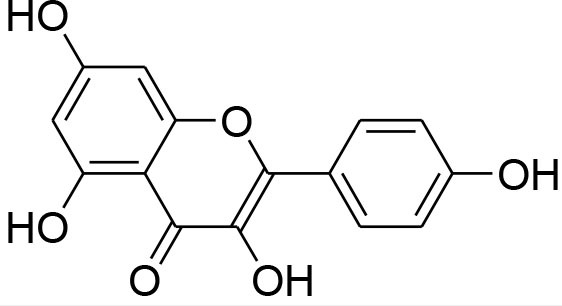	Down regulated expression and activity of MMP-2/-9 inMDA-MB-231 cell line by repressing MAPK signaling pathway	30 μM	Gelatin zymography RT-PCR analysis Western blotting EMSA	Li et al., 2015
Luteolin-8-C-β-fucopyranoside	*Anthraxonhispidus*	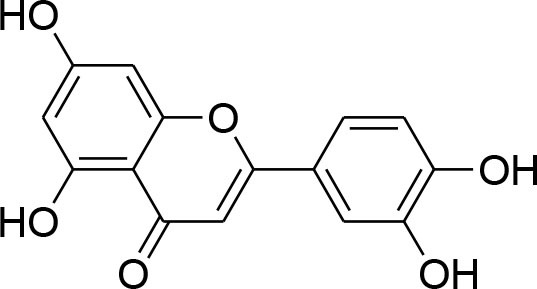	Suppressed TPA-provoked MMP-9 secretion and mRNA expression via inhibition of MAPK signaling pathway nuclear AP-1 and NF-κBdown regulation	6.9 μM	Gelatin zymography RT-PCR analysis Western blotting EMSA Immunohistochemistry	Park et al., 2013
Orientin luteolin 8-C-β-D-glucopyranoside	*Anthraxonhispidus*	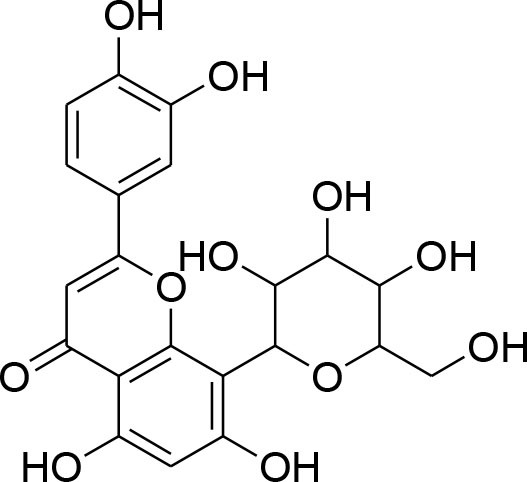	Inhibition of MMP-9 via inhibiting TPA-instigatedactivation of PKCα and ERK, along with nuclear translocation of STAT3 and AP-1		Gelatin zymography RT-PCR analysis ELISA Western blotting Immunofluorescence	Kim et al., 2018a
7-Methoxy-luteolin-8-C-β-6-deoxy-xylo-pyranos-3-uloside (mLU8C-PU)	*Anthraxonhispidus*	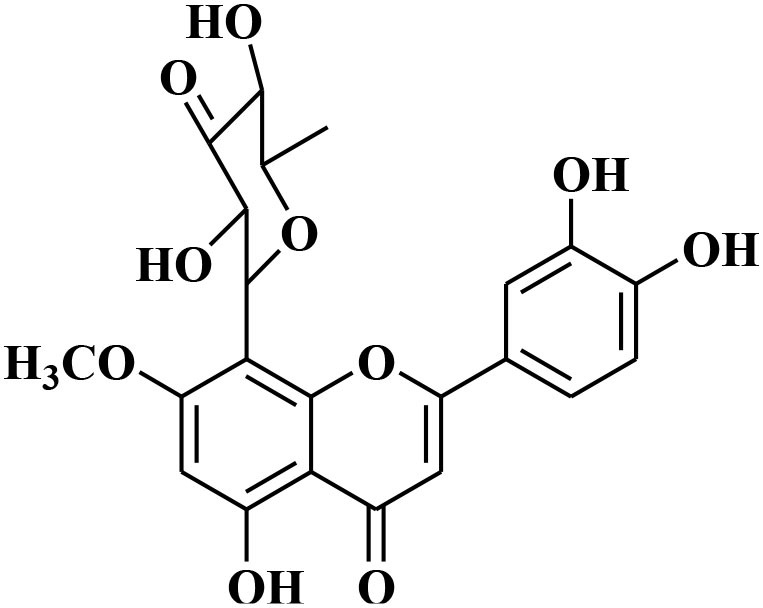	Suppression of MMP-9 expression By mitigation of TPA-provokedactivation ofPKCα, JNK, and the nuclear translocation of AP-1 and NF-κB.		Gelatin zymography, RT-PCR analysis ELISA Western blotting Immunofluorescence	Kim et al., 2018b
Silibinin	*Silybummarianum*	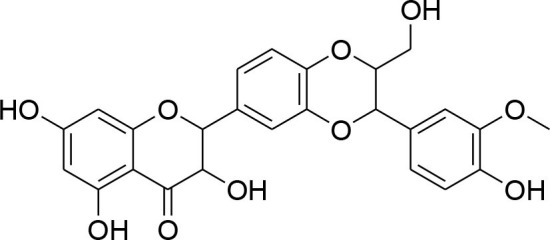	Arresting TPA-provoked MMP-9 expression via suppressing Raf/MEK/ERK pathway	207 μg/ml	midruleGelatin zymography, Western blotting ELISA	Lee W. Y. et al., 2007
			Inhibition of PMA-instigated expression of MMP-9 by suppressing activation of AP-1 via MAPK signaling pathway		Gelatin zymography Transient transfection and luciferase reporter assay EMSA Western blotting	Kim S. et al., 2009
Anthocyanins	*Oryza sativa*	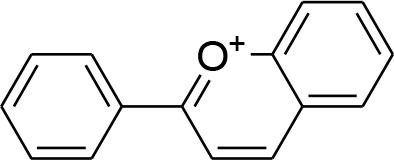	Repression of MMP-9 expression	32.1 μg/ml	Gelatin zymography	Chen et al., 2006
Turmerone	*Curcuma longa*	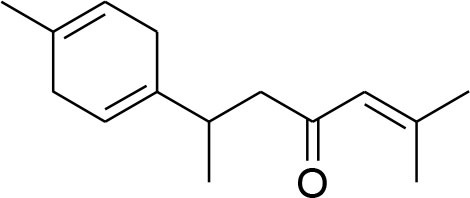	Attenuation of invasion and MMP-9 expression by Inhibiting NF-kB activation in TPA-Induced Breast Cancer Cell	243 μM	Gelatin zymography Western Blotting RT-PCR analysis Immunofluorescence confocal microscopy Chromatin immunoprecipitation assay Transient transfection and dual luciferase assay	Park et al., 2012
Baicalein	*Scutellariabaicalensis*	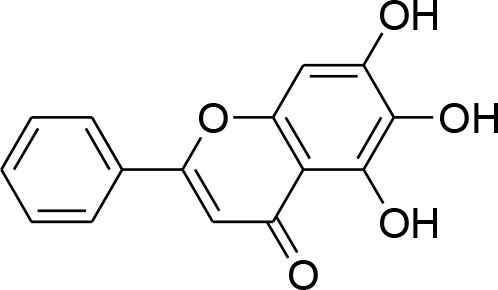	Attenuation of expression and secretion of MMP-2 and 9		Gelatin zymography Western Blot analysis	Wang et al., 2010
Berbamine	*Berberisamurensis*	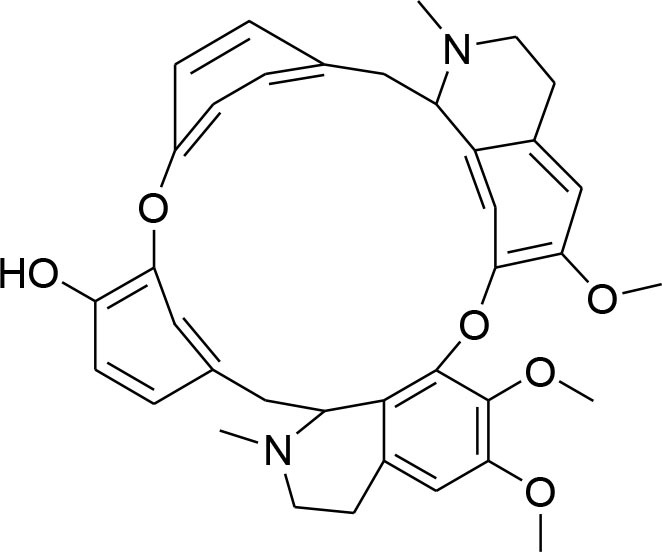	Suppression of activation and expression of pro-MMP-9/-2	23.75 μM	Gelatin zymography RT-PCR analysis	Wang et al., 2009
Daidzein		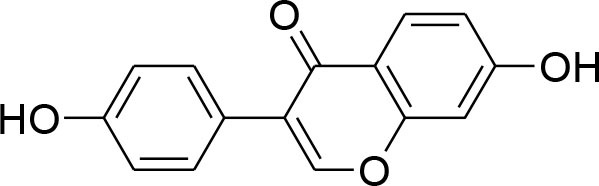	Inhibition of invasion of MDA-MB-231 human breast cancer cells in part via the down-regulation of MMP-2 expression	59.7 μM	RT-PCR analysis	Magee et al., 2014
Delphinidin	*Punicagranatum*	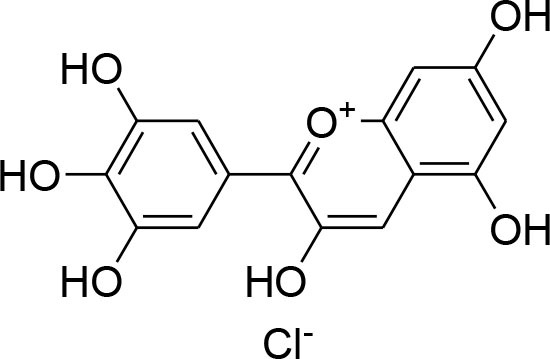	Suppression of PMA-provoked expression of MMP-9 via blockage of activation of NF-κBby MAPK Signaling Pathways	120 μM	Gelatin zymography Western blotting RT-PCR analysis Matrigel invasion assay	Im et al., 2014
Gingerol	*Zingiberofficinale*	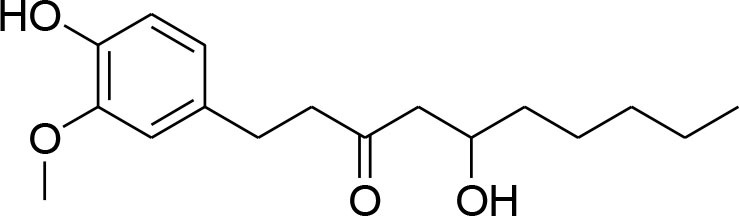	Reduced mRNA expression level of MMP-2 and 9	205 μM	Gelatin zymography RT-PCR analysis	Lee et al., 2008
Isoliquiritigenin	*Glycyrrhizaglabra*	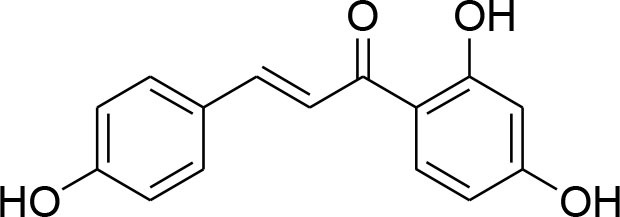	Reduced mRNA expression level of MMP-2 and 9	32.66 μM	Gelatin zymography Western blotting	Wang et al., 2013
Morin	*Ficuscarica*	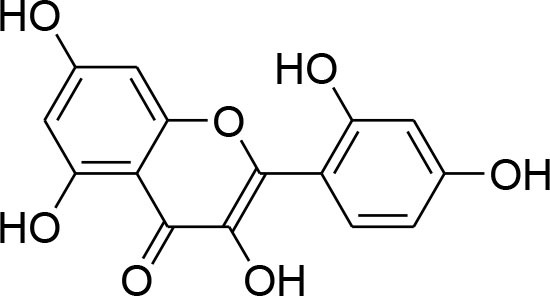	Reduced mRNA expression level of MMP-9	7.82 μM	Gelatin zymography	Jin et al., 2014
Myricetin	*Azadirachtaindica*	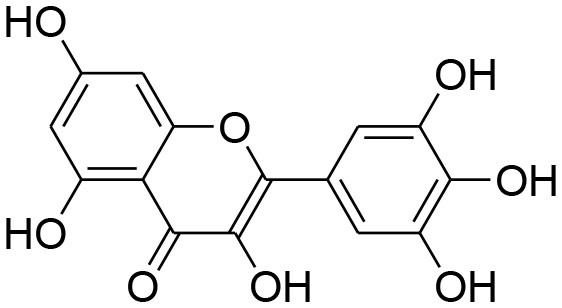	Reduced mRNA expression level of MMP-2 and 9	2.7 μg/ml	Western blotting RT-PCR analysis	Ci et al., 2018
Oleuropein	*Oleaeuropaea*	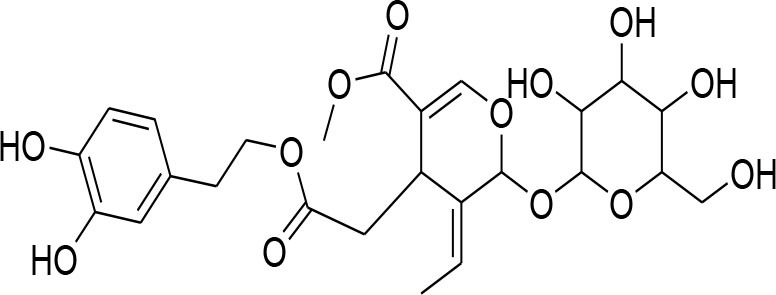	Overexpression of TIMP-1, 3 and 4 along with downregulation of MMP-2 and 9 gene expression		RT-PCR analysis	Hassan et al., 2012
Quercetin		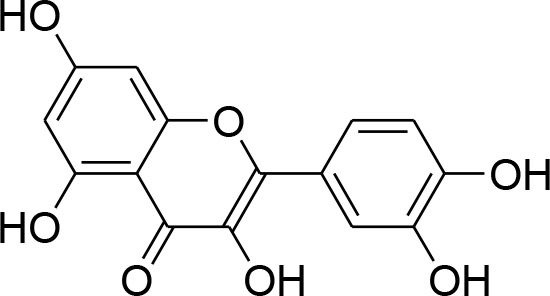	Suppression of PKCd/ERK/AP-1-dependent matrix metalloproteinase-9 activation	40.2 μM/ml	Gelatin zymography RT-PCR analysis Western blotting	Lin et al., 2008
**Lignans**						
Arctigenin	*Arctiumlappa*	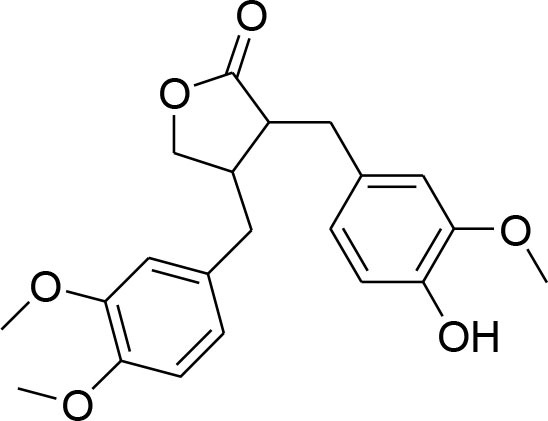	Suppression of enzyme activity of MMP-2/-9		Gelatin zymography Western blotting	Lee W. Y. et al., 2007
			Down regulation of MMP-2/-9			(Lou et al., 2017)
enterolactone	*Linumusitatissimum*	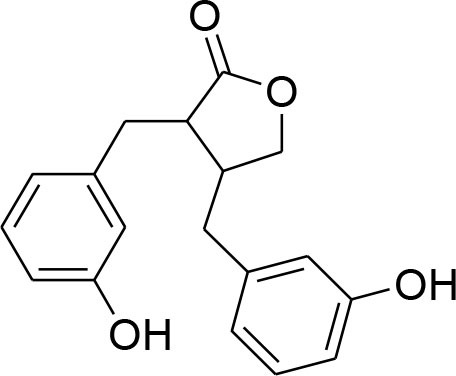	MMP-2/-9 expression downregulation	54.33 μM	RT-PCR analysis Gelatin Zymography	Mali et al., 2012
			Reduced proteolytic activity of MMP-2/-9			(Mali et al., 2017)
**Terpenoids**						
Ursolic acid	*Maluspumila*	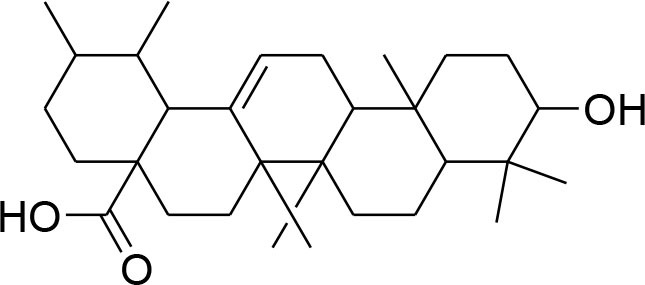	Down regulation of MMP-2 via inhibition of Jun N-terminal kinase and Akt Over expression of TIMP-2	20 μM	Gelatin Zymography RT-PCR analysis Western blotting	Yeh et al., 2010
Eusaphic acid	*Eriobotrya japonica* Lindley		Inhibition of MMP-2 and MMP-9 activities		Gelatin zymography	Kim M. et al., 2009
Tanshinone II	*Salvia miltiorrhiza Bunge*	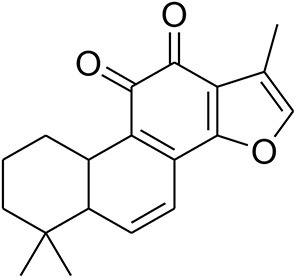	decrease expression of MMP-7 and ETS transcription factors		MTT	Wang et al., 2005
Antroquinonol	*AntrodiaCamphorata*	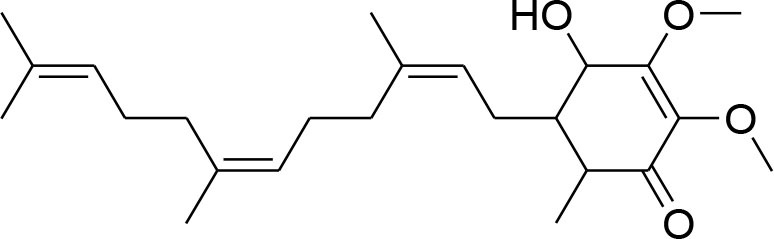	inhibition of MMP-9 gene expression and enzyme activity via inhibiting ERK-AP-1- and AKT-NF-κB	10 μM	Western Blotting Gelatin zymography RT-PCR assay	Lee et al., 2015
Carnosol	*RosmarinusOfficinalis*	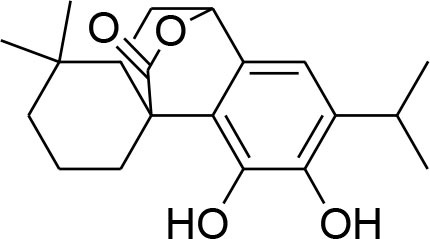	diminish activity of MMP-9		Gelatin zymography	Iratni et al., 2014
Pachymic acid	*PoriaCocos*	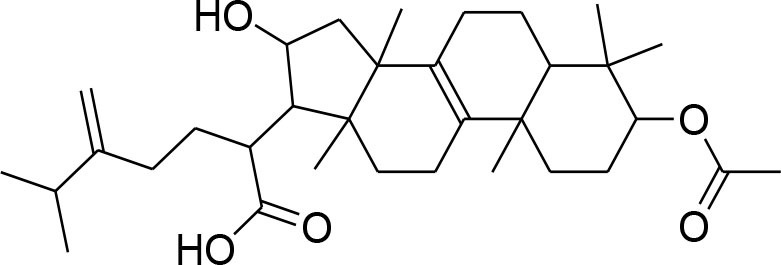	decrease MMP-9 secretion by reducing MPA-induced transcriptional activity of NF-kB	0.26 μM	Gelatin zymography, RT-PCR analysis Luciferase reporter assay	Ling et al., 2011
Oleandrin	*Nerium Oleander*	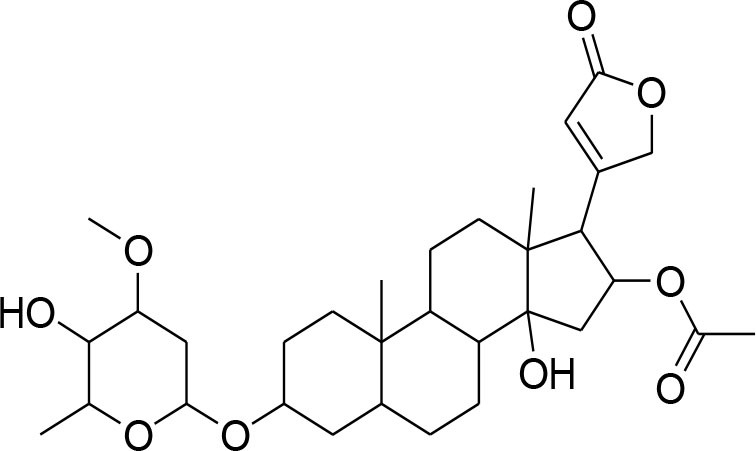	reduced MMP-9 activity by downregulation of STAT-3 phosphorylation		Gelatin zymography, western blotting	(Ko et al., 2018)
**Glycosides**						
Picroside II	*PicrorrhizaKurroa*	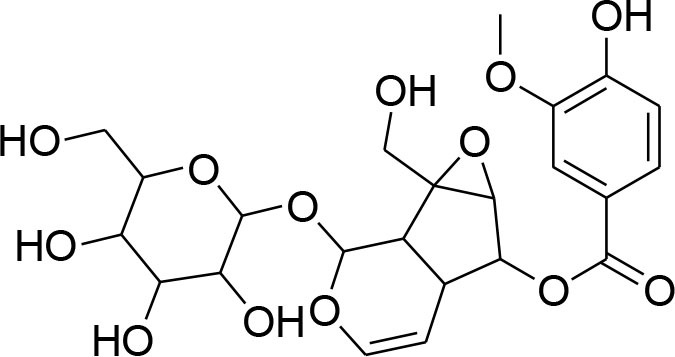	Inhibiting activity of MMP-9 in MDA-MB-231 cancer cells	61.86 μg/ml	Gelatin zymography, Immunohistochemistry	Lou et al., 2019
Picroside I, Kutkoside, Kutkin	*Picrorrhizakurroa*	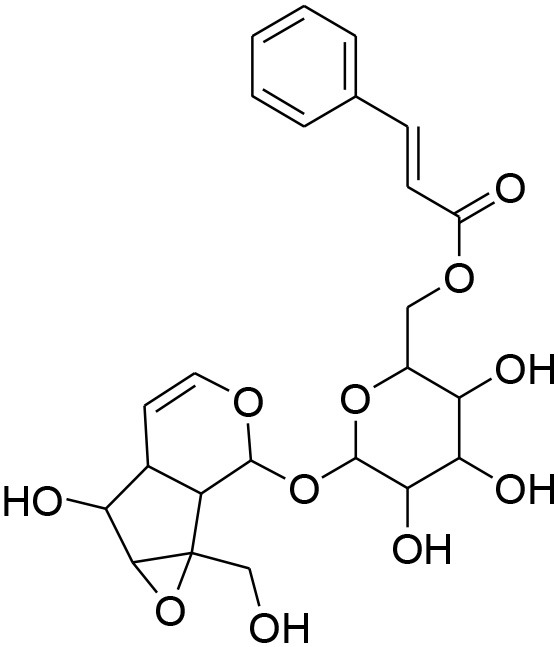	Downregulation of MMP-2,9,1,13	61.86 μg/ml	Gelatin zymography RT-PCR analysis	Rathee et al., 2013
		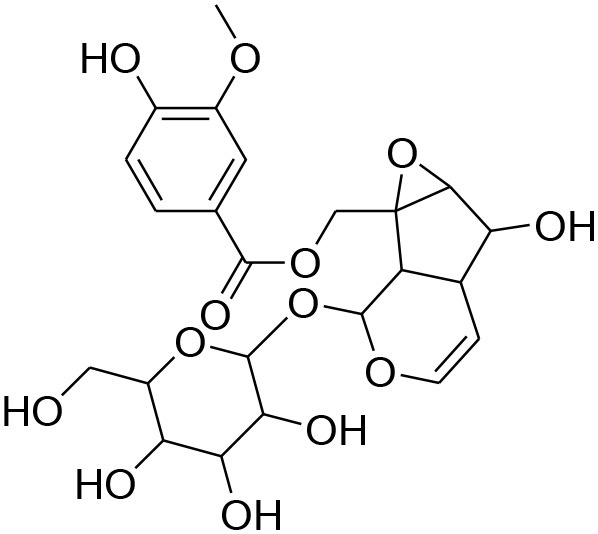				
		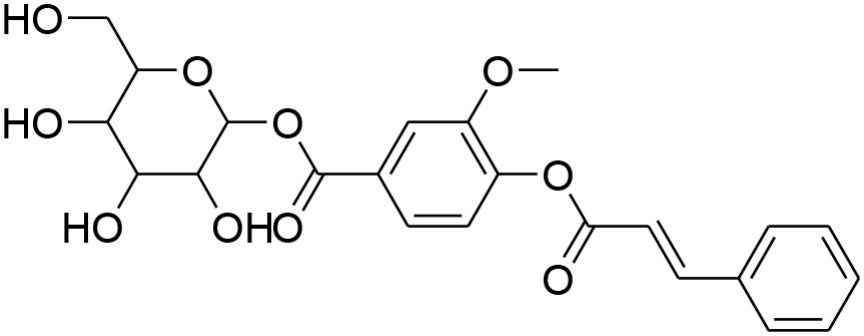				
Luteolin	*Arthraxonhispidus*	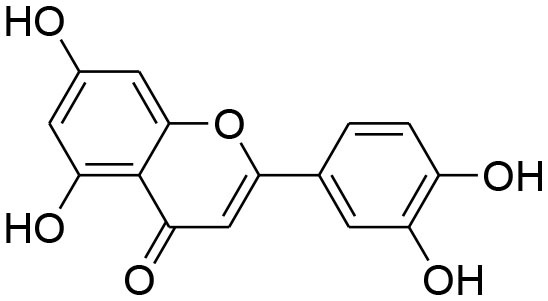	Inhibition of MMP-9		RT-PCR analysis	Lee et al., 2019

## Phytochemicals Modulating MMPs Activation and Associated Signaling Pathways

### Alkaloids

Alkaloids are documented to be the diverse class of natural compounds bearing a ring structure with nitrogen atom (Lu et al., 2012). Several alkaloids exhibit proven pharmacological attributes including ephedrine's pain relieving potential for asthmatics, morphine's analgesic attribute, and vinblastine's anticancer attributes (Benyhe, 1994; Li W. et al., 2007; Lee, 2011). Alkaloids are considered to be highly active constituents in natural flora, and some of them have previously been effectively established into anti-cancer drugs such as camptothecin (CPT), a well-known inhibitor of topoisomerase I (TopI) (Huang et al., 2007) and vinblastine, which produces its anti-cancerous impact via interacting with tubulin (Li W. et al., 2007).

Berberine, a quaternary ammonium isoquinoline alkaloids, can be found mostly in plant species belonging to the *Berberis* genus (Bashar et al., 2014). Berberine possesses an array of biological activities. Diversified studies have pointed out anti-tumorigenic potential of berberine by virtue of *in vitro* and *in vivo* experimental techniques. The evaluation of structure activity relationship (SAR) of substituents at R position of berberine revealed that their derivative possesses better cytotoxic potential as compared to parent compound. Of 20 newly synthesized compounds, 9-O-[4-ethyl-1-(naphthalene-2-ylmethyl)-1H-1,2,3,triazolel berberrubine chloride exhibits optimal anticancer activity against human breast cancer cell MCF-7 with (IC_50_ = 41.536 ± 3.4 um). The increased anticancer activity of berberine derivative is due to the activation of tumor necrosis factor alpha (TNF-α) which in turn produces substantial increase in MMP-9 activity. This elevated expression of TNF-α and MMP-9 as well as MMP-9 activity is promisingly attenuated by Berberine (Kim et al., 2008; Jin et al., 2015). TNFα-induced activator protein-1 (AP-1) DNA binding activity is also inhibited by berberine. Moreover, berberine can cause significant reduction in TNFα-provoked cell invasion. Taken together, TNF-α provoked MMP-9 activation leading to cell invasion is observed to be reduced by berberine via suppressing activity of AP-1 DNA (Kim et al., 2008). AP-1 is a transcriptional factor of MMP-9 and as such, suppression of AP-1 DNA binding activity can lead to suppression of MMP-9. It is important to note that MMP-9 secretion is elevated in several types of human cancer, and such elevation is correlated with poor prognosis (Roomi et al., 2009). However, another study concluded that berberine induced suppression of MMP-2 and MMP-9 involved the inhibition of the Akt/nuclear factor kappa B (NF-kappaB) and activator protein-1 (AP-1) signaling pathways (Kuo et al., 2012).

Evodiamine, a major bioactive quinolone alkaloid isolated from *Evodiarutaecarpa* (Lu et al., 2012), proves its anti-cancerous potential in both *in vitro* and *in vivo* models by arresting cell cycle, inducing apoptosis, preventing angiogenesis, invasion, and metastasis in a variety of cancer cell lines (Ogasawara et al., 2001, 2002; Fei et al., 2003; Zhang et al., 2003). The derivatives become more cytotoxic after an appropriate substitution on N13-position. Higher antitumor and broad spectrum activity has been observed by the one with a benzoyl group at N13-position against MDA-MB-435 cells (Dong et al., 2010). Evodiamine derivative promisingly inhibits cell migration and invasion via down regulation of MMP-9, urokinase-type plasminogen activator (uPA), and uPAR expression. Moreover, evodiamine down regulates p-ERK and p-p38 MAPK expression (Du et al., 2013).

Matrine exhibits various pharmacological properties including antiviral, antibacterial, anti-inflammatory, antiarrhythmic, anti-asthmatic, anti-obesity, diuretic, anticancer, choleretic, hepatoprotective, and cardioprotective effects (Lu et al., 2012). Matrine significantly attenuates tumor invasion, activation of MMP-9/MMP-2, Akt phosphorylation, DNA binding activity, nuclear factor-κB expression and mRNA levels of MMP-9, MMP2, EGF, and VEGFR1 in MDA-MB-231 cells. Matrine thus inhibits cancer cell proliferation and invasion via EGF/VEGF-VEGFR1-Akt-NF-κB signaling pathway (Yu et al., 2009).

Piperine, a piperidine alkaloid isolated from *Piper nigrum* and *Piper longum*, is a compound found in famous spices that have been used for centuries. Piperine possesses anti-inflammatory, antioxidant, antidiarrheal, antimutagenic, anticonvulsant, hypolipidemic, and tumor inhibitory activities (Lu et al., 2012). Piperine inhibits PMA-induced MMP-9 expression through down regulation of PKCα/extracellular signal-regulated kinase (ERK) 1/2 and reduction of NF-κB/AP-1 activation (Lai et al., 2012; Do et al., 2013).

Sanguinarine, isolated from family papaveracea, is a benzophenanthridine with proven biological activities including antifungal, antibacterial, antiplatelet, antischistosomal, anti-cancerous, and anti-inflammatory. It has been used extensively for controlling schistosomiasis (Lu et al., 2012). Sanguinarine displays its anti-invasive impact on MDA-MB−231breast cancer cell lines via tightening the tight junctions (TJs) and reducing mRNA expression of MMP-2/-9 (Choi et al., 2009).

### Flavonoids

Flavonoids are hydroxylated phenolic plant components, produced in response to microbial infection. Flavonoids have provoked colossal awareness in the earlier decade by virtue of their multifaceted health effects in humans and animals (Karak, 2019). They are termed as “functional components” and “health supporting biomolecules” due to their probable role in upholding health and averting the chronic deteriorating diseases (Nijveldt et al., 2001). Flavonoids are subdivided into different groups commonly flavones, flavonols, flavanones, flavanols, anthocyanins, and isoflavones.

Quercetin, a natural dietary flavonoid is obtained from barks of many plants, vegetables, fruits and employs antioxidant, anticancer, and antiinflammatory properties. Methyl capping of one or more free hydroxyl group improve compound cytotoxicity e.g., 3-*O-*methylquercetin and tamarexetin exhibited increased cytoxicity (Phatak, 2016). They control p38 MAPK signaling that persuade apoptosis in MCF-7 and MDA-MB-231 breast carcinoma cells (Ranganathan et al., 2015). Moreover, Quercetin derivatives significantly down-regulate Akt/MMP-9 pathway and suppress PKCd/ERK/AP-1-dependent matrix metalloproteinase-9 activation, thereby proved its anti-MMP activity confirmed through western blotting, gelatin zymography and RT-PCR analysis (Lin et al., 2008).

Kaempferol, a dietary flavonoid present in edible plants (beans, kale, tea, endive, broccoli, tomato, cabbage, and grapes) has been used as anticancer, antimicrobial, antioxidant, cardioprotective, neuroprotective, and anti-allergic compound (Kim and Choi, 2013). Kaempferol averts breast tumor infiltration and progression in MDA-MB-231 breast carcinoma cells by reducing MMP-2 and 9 expressions and subdual of AP-1 and MAPK signaling (Kim et al., 2016).

Delphinidin, found primarily in pomegranate extract, is consumed as dietary supplement. The presence of ortho-dehydroxyphenyl structure on β-ring and a free hydroxyl group at position-3 is responsible for highest inhibitory potency (Tang et al., 2015). A latest study explained that delphinidin treatment represses cell propagation and persuades apoptosis in triple-negative, ER-positive and HER2-overexpressing breast tumor cell lines, devoid of any toxic outcomes in normal breast cells (Ozbay and Nahta, 2011). In addition, MAPK signaling is repressed in both triple-negative and ER-negative breast carcinoma cells but not in MCF-10A normal cells. Delphinidin inhibits Ras-ERK MAPKs and PI3K/AKT/mTOR/p70S6K pathways (Hertog et al., 1993). Delphinidin also possess anti-angiogenic and anti-invasive properties by diminishing MMP-9 activity in ER+ MCF-7 cells by blocking NF-κB via MAPK signaling pathways (Im et al., 2014).

Daidzein, an isoflavonoid that can be isolated from various plants and herbs, has proven antiproliferative impact on breast cancer cells via inhibiting TNF-α expression, down-regulation of MMP-9 mRNA expression, as evident through RT-PCR analysis (Magee et al., 2014).

Genistein, isolated from soybean possesses chemopreventive potential against various cancer cells including prostate and breast cancer. Genistein has proven inhibitory potential against breast cancer cell proliferation, growth, invasion, and metastasis in various *in vitro* and *in vivo* models (Hsieh et al., 1998). NF-κB signaling pathway plays an imperative role not only in angiogenesis but also in cell growth, inflammation, apoptosis, and invasion. Genistein treatment considerably subdued MMP-9 activation by NF-κB signaling inactivation (Gupta et al., 2010). Genistein also displays down-regulation of all MMP genes (MMP-1,−2,−3,−7,−9,−11,−14,−15,−16) along with up-regulation of TIMP-1 level while TIMP-2 level is being suppressed in MDA-MB-231 and MCF-7 cancer cell lines (Kousidou et al., 2005; Lee W. Y. et al., 2007).

### Lignans

Lignans or phytoestrogens constitutes a group of natural compounds that manifest biological attributes via interfering with estrogen metabolism. This interference in estrogen metabolism is produced due to estrogenic as well as anti-estrogenic properties of lignans. Daily supplementation of lignans by females has been associated with hormonal changes that are favorable for women of all ages. Owing to reported benefits, lignans gained considerable attention as chemotherapeutic agent (Ezzat et al., 2018). Pre-clinical studies have been conducted to explore the anti-cancerous potential of lignans and reported their benefits in suppressing the early tumorigenesis (Landström et al., 1998; Bylund et al., 2000) along with actions on tumor growth inhibition, angiogenesis, and disease progression (Jungeström et al., 2007; Lin et al., 2008). Flaxseeds are thought to be the richest source of lignans and contain secoisolariciresinoldiglucoside (SDG), which is a major lignan possessing proven health benefits such as cardio protection, antioxidant, and anticancer effects (Alphonse and Aluko, 2015). SDG in mammalian body is metabolized to Enterodiol (ED) and Enterolactone (EL) by intestinal bacterias (Fuentealba et al., 2014). These mammalian lignans derived from SDG have shown promising benefits in breast cancer (Flower et al., 2014). The α-linolenic acid (ALA) is a precursor of PUFA omega 3 family. Eicosapentaenoic acid (EPA) and docosahexaenoic acid (DHA) are formed by PUFA omega-3. ALA and omega 3 fatty acid are main constituents of flaxseed which possess anti-cancer property (Calado et al., 2018). Mammalian lignans have structure similarities to the estrogen with significant antioxidant potential but weak estrogenic action (Calado et al., 2018). Findings of RT-PCR and gelatin zymography assay show that SDG metabolite entereolactone significantly downregulates mRNA expression of MMP-9 and MMP-2 (Mali et al., 2012). Arctigenin, another natural lignan, derived from *Arctium lappa* also demonstrates inhibitory potential against MMP-9 and MMP-2 confirmed through western blotting and gelatin zymography (Lou et al., 2017). Taken together, lignans can be considered as useful natural remedies against breast cancer by suppressing MMP- activity, thereby helpful in preventing ECM degradation.

### Glycosides

Glycosides comprise a large group of secondary metabolites that are widely distributed in plants. These have been employed as dietary agent or complementary medicine for management and treatment of cancer (Khan et al., 2019). The anti-metastatic effects of Picroside II, an iridoid glycoside derived from a high valued medicinal herb *Picrorhiza Kurroa*, was assessed on human breast cancer MDA-MB-231 cells with a focus on matrix metalloprotenaise (MMP) activity. Zymography experiments showed that the Picroside II glycosides led to suppression of MMP-9 activity in MDA-MB-231 cells in dose dependent manner (Lou et al., 2019).

*Picroside Kurroa* extract and its isolated iridoid glycosides Kutkin, Kutkoside and Picroside I exhibit cytotoxic potential in a dose dependent manner. This cytotoxic activity is primarily attributed to downregulation of gelatinases (MMP-2 and MMP-9) and collagenases (MMP-1 and MMP-13), decreased expression of MMPs at mRNA and protein level, inhibited migration, and invasion of human breast MCF-7 cells as detected by gelatin zymography and RT-PCR (Rathee et al., 2013).

A study showed that leuteolin glycosides, obtained from Arthraxonhispidus, have anti-metastatic activity through suppression of MMP-9 and inhibition of invasion and migration in MDA-MB-231 cells (Lee et al., 2019).

Oleandrin and odosroside are extracted from the Nerium oleander. These compounds comprise of lactone ring at carbon position 17 (C17) and a steroidal nucleus linked with sugar at position 3 (C3). A study has revealed reduction in mortality among breast cancer patients following the treatment with these compounds (Pongrakhananon, 2013). Oleandrin suppresses MMP-9 activity and octamer binding transcription factor 3/4 (OCT3/4) through inhibition of phosphor-signal transducer and activator of transcription (STAT)-3 at 50 and 100 nM doses, respectively (Ko et al., 2018).

### Terpenoids

Terpenoids, also known as isoprenoids, are the most numerous and structurally diverse natural products found in many plants. Several studies, *in vitro*, preclinical, and clinical have confirmed that this class of compounds displays a wide array of very important pharmacological properties. This diverse class has variety of functional roles in the field of food, medicine and cosmetics. Existing evidence suggests thee anticancer potential of terpenoids attributing to their inhibitory actions on cancer cells (Perveen and Al-Taweel, 2018).

Ursolic acid (UA) is natural triterpinoid obtained from apple (Malluspumila) peel and has potential to prevent and treat breast cancer. N-acylimidazole and N-alkylimidazole derivatives of UA with α,β-unsaturated ketone exhibit significant anti-cancer activity (Chen et al., 2015). Migration and invasion of highly metastatic MDA-MB-231 cells have been suppressed by dose and time dependent effect of urosolic acid derivatives. The down-modulation of MMP-2 and uPA expression is associated with inhibition of JNK-Akt and reduction of NF-Kb p65 in nucleus (Yeh et al., 2010).

Loquat extract (*Eriobotrya japonica*) has found its clinical application in reducing breast cancer metastasis and invasion. A study showed that its leaf extract has more potent chemopreventive effect than seed extract. The findings from gelatin zymographic assay reveal that its leaf extract inhibits the growth of MDA-MB-231 and activities of MMP-2 and MMP-9 more significantly than seed extract (Kim M. et al., 2009).

Tanshinone IIA (Tan IIA) is a member of the major lipophilic components extracted from the root of *Salvia miltiorrhiza Bunge*, which is currently used in China and other neighboring countries to treat patients suffering from myocardial infarction (MI), angina pectoris, stroke, diabetes, sepsis, and other conditions. It has anti-oxidant and potential cytotoxic activities. Tanshinone IIA suppresses MMP-7 and epithelium specific (ETS) transcription factors involved in tumorigenesis relevant to human breast cancer cells (Wang et al., 2005).

The ubiquinon derivative isolated from *Antrodia Camphorata* is Antroquinonol. It has been found to have anti-tumor effect by suppressing ERK-AP-1 and AKT-NF-kB dependent MMP-9 activity (Lee et al., 2015).

A natural constituent derived from *Rosmarinus officinalis* is carnosol which has anti-carcinogenic and antioxidant properties. The invasion and migration of B16/F10 cells is significantly inhibited by carnosol. Furthermore, carnosol suppresses MMP-9 expression through inhibition of extracellular signal regulated kinase (ERK) 1/2, AKT, and JNK signaling pathway (Huang et al., 2005).

## Conclusion

Matrix metalloproteinases (MMPs) supports cancer invasion not only by degradation of basement membrane but also through release of some factors supporting cancer cell growth and preventing apoptosis. Several clinically approved MMP inhibitors have MMP-modulating potential but have limited use amid associated unwanted effects. The suppression of MMPs during cancer metastasis proves beneficial which can be done by blocking their activation or activity at transcription level via interfering with extracellular factors and signal transduction pathways (i.e., NF-kB or AP-1). Herbal medications have gained much attention during the past decades in healthcare systems. Phytochemicals derived from plant sources are a great source for novel drug development due to their safety and efficacy. These secondary metabolites bear the potential of modulating different pathological pathways responsible for number of disorders. Current review underscores the potential implications of phytochemicals in the management of breast cancer via direct inhibition of MMPs activation or by modulation of signaling pathways associated with MMPs activation. These phytoconstituents can serve as useful modalities for prevention of breast cancer invasion and metastasis. Natural MMPs inhibiting agents are now used for the development of alternative therapeutic strategies for treatment of breast cancer as they complement the action of conventional treatment strategies through their multi-targeting capacity. Modulation of MMPs is an emerging and promising area of research which could be translated to develop new therapeutic options for various subtypes of breast cancer.

## Author Contributions

AU, YK, SQ, and MR: conception or design of the work. NA, TM, AU, and AIA: analysis or interpretation of data for the work. YK, AU, SQ, and NA: drafting the work. TM, MI, MR, S-U-DK, and ASA: revising the manuscript critically for important intellectual content. All authors: provided approval for publication of the content and are accountable for all aspects of the work.

## Conflict of Interest

The authors declare that the research was conducted in the absence of any commercial or financial relationships that could be construed as a potential conflict of interest.
